# Influence of Elevated Temperature on the Mechanical Properties of Hybrid Flax-Fiber-Epoxy Composites Incorporating Graphene

**DOI:** 10.3390/polym14091841

**Published:** 2022-04-29

**Authors:** Amer Oun, Allan Manalo, Omar Alajarmeh, Rajab Abousnina, Andreas Gerdes

**Affiliations:** 1 Centre for Future Materials, Faculty of Health, Engineering and Sciences, University of Southern Queensland, Toowoomba 4350, Australia; allan.manalo@usq.edu.au (A.M.); omar.alajarmeh@usq.edu.au (O.A.); rajab.abousnina@mq.edu.au (R.A.); 2 KIT Innovation Hub, Department of Civil Engineering, Geo and Environmental Sciences, Karlsruhe Institute of Technology, 76131 Karlsruhe, Germany; andreas.gerdes@kit.edu

**Keywords:** elevated temperature, flax fiber, natural fiber composite, mechanical properties, graphene nanoparticles, SEM

## Abstract

Natural fibers are now becoming widely adopted as reinforcements for polymer matrices to produce biodegradable and renewable composites. These natural composites have mechanical properties acceptable for use in many industrial and structural applications under ambient temperatures. However, there is still limited understanding regarding the mechanical performance of natural fiber composites when exposed to in-service elevated temperatures. Moreover, nanoparticle additives are widely utilized in reinforced composites as they can enhance mechanical, thermal, and physical performance. Therefore, this research extensively investigates the interlaminar shear strength (ILSS) and flexural properties of flax fiber composites with graphene at different weight percentages (0%, 0.5%, 1%, and 1.5%) and exposed to in-service elevated temperatures (20, 40, 60, 80, and 100 °C). Mechanical tests were conducted followed by microscopic observations to analyze the interphase between the flax fibers and epoxy resin. The results showed that a significant improvement in flexural strength, modulus, and interlaminar shear strength of the composites was achieved by adding 0.5% of graphene. Increasing the graphene to 1.0% and 1.5% gradually decreased the enhancement in the flexural and ILSS strength. SEM observations showed that voids caused by filler agglomeration were increasingly formed in the natural fiber reinforced composites with the increase in graphene addition.

## 1. Introduction

Synthetic-fiber-based polymer composites are now being used in a number of industrial applications including civil engineering and construction, packaging, and automotive industries due to their high strength, stiffness, and durability. However, the manufacturing and use of synthetic fiber composites have some detrimental effects on the environment due to their nondegradable nature, reduced recyclability, and toxicity [1]. Moreover, manufacturing of synthetic fibers such as glass, carbon, and aramid consumes more energy compared with that of natural fibers [2]. For example, the energy needed for flax fiber production is 9.55 MJ/kg while for glass fiber it is 54.7 MJ/kg, as reported by Joshi et al. [3] and Balla et al. [4]. The associated problems with the use of artificial fibers in manufacturing polymeric composites have led to an increased interest in plant-based fibers [5,6]. Using natural fibers can also result in high-performance composites [7] and renewable composites [8] at a relatively low cost [4]. Natural-fiber-based composites are now being used for automotive parts, construction elements [9], and wind turbine blades [10]. One major reason behind the increasing attraction of using natural fibers is their renewable nature, which can address the production instability issue of synthetic fibers associated with the shortage of oil resources [11,12]. Moreover, natural fiber composites can result in new materials that will not add to the growth of waste and promote sustainability [13]. Thus, interest has increased in exploring the use of different types of natural fibers as new reinforcements in polymeric composites.

Among many different types of plant fibers, flax is the most commonly used fiber for manufacturing natural fiber composites. Flax fibers are extracted from bast, which has a high cellulose percentage (approximately 30–76% by weight) as well as a low microfibril angle of 2 to 8 degrees. The cellulose percentage is responsible for the strength of the fibers while the microfibril angle controls the stiffness. Both contribute to the mechanical properties of the fibers and the performance of the composites [4]. Xiong et al. [14] stated that the addition of woven flax fibers into polyoxymethylene (POM) matrix improved the properties of flexural strength and tensile strength of POM composites compared with the control specimens. Prasob and Sasikumar [8] also indicated that polymer composites incorporating flax fibers and nanofillers have the same mechanical strength as glass-fiber-based polymer composites. Wang et al. [15] found that flax-based epoxy grafted with nanoparticles can significantly improve the flexural properties of the composites. In another investigation, Foruzanmehr et al. [16] observed that the graft of nanofillers can significantly enhance both the mechanical and physical properties of flax and polylactic acid (PLA) matrix nanocomposites. Nabinejad [17], on the other hand, indicated that the hybridization of natural fiber composites with nanoparticles requires more attention and suggested that further investigation is needed to determine the effects of nanofillers such as graphene nanoparticles on the mechanical properties of hybrid composites.

One limitation of natural fibers as effective internal reinforcements in polymeric composites is their lower thermal degradation property when compared with synthetic fibers [4]. This property of natural fiber composites restricts their widespread use in industrial and engineering applications. Many researchers have highlighted the sensitivity of synthetic-based fiber-reinforced polymer (FRP) composites at in-service elevated temperatures, due to the decomposition processes and glass transition of the polymer matrix material [18]. Although the structural performance of FRP composites at room temperature is considered acceptable, the behavior of composite materials under elevated temperatures is complex—a factor that Manalo et al. [19] highlighted still has limited awareness and requires a more detailed investigation. A better understanding of the behavior of natural fiber composites filled at in-service elevated temperature is therefore crucial for their wide acceptance and use in mechanical and structural engineering applications.

A number of investigations have evaluated the performance of synthetic-based FRP composites with and without nanofillers at in-service elevated temperatures under different loading conditions including tension, compression, interlaminar shear (ILSS), and flexure [8,20,21,22,23,24,25]. These researchers have concluded that the exposure of FRP composite materials to elevated temperatures resulted in a decrease in the mechanical properties but at different magnitudes depending on the type of resin systems, fiber reinforcements, and nanofillers. Moreover, the degradation of mechanical properties can be related to their glass transition temperature, which mainly depends on the type of resin used. However, the strength retention of polymer against in-service elevated temperature can be improved with the addition of particulate fillers [26,27]. Thus, the effects of nanofillers on the behavior of natural fiber composites under in-service temperatures require a more detailed consideration.

This study investigates for the first time the effectiveness of graphene nanoparticles in improving the performance of flax-fiber-based natural composites at in-service elevated temperatures. The experimental investigation focuses on the flexural and interlaminar shear performance of hybrid flax-reinforced epoxy-based composites with different levels of graphene by weight (0, 0.5, 1.0, and 1.5%) and tested at different levels of elevated temperature (RT, 40, 60, 80, and 100 °C, where RT is the room temperature of 20 °C). The results of this study will provide comprehensive information on the effect of graphene addition on natural fiber composites and their performance at in-service elevated temperatures. Moreover, it will support the development and application of cost-effective and eco-friendly biocomposites for industrial and engineering applications.

## 2. Experimental Program

### 2.1. Materials

Epoxy resin (R246TX) mixed with hardener (H160 Kinetix Medium Hardener) supplied by ATL Composites, Molendinar, Australia was used as a matrix in the manufacturing of the composites. The resin to hardener ratio used was 1:4 by weight, as recommended by the supplier. Graphene nanoparticles with average specific area of 300 m^2^/g and elastic modulus of 340 GPa were supplied by Sigma-Aldrich, Castle Hill, Australia. The unidirectional flax fibers with density of 200 g/m^2^ were supplied by Colan Composite Reinforcement, Huntingwood, NSW, Australia. Table 1 lists the properties of neat epoxy resin, natural fibers, and graphene based on the available literature and as provided by the supplier.

### 2.2. Specimen Preparation

**Figure 1:**
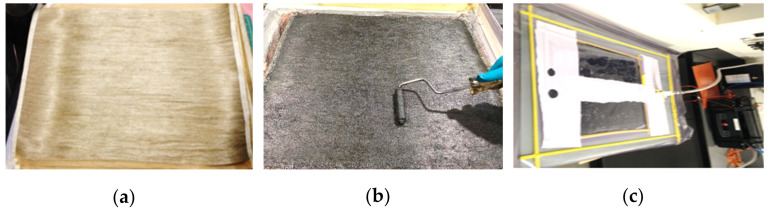
(**a**) Unidirectional flax fibers. (**b**) Wetting the fibers. (**c**) Vacuum bagging.

The flax fibers were arranged in a longitudinal direction and prepared as a sheet with dimensions of 600 mm in length and 400 mm in width (see Figure 1a). These sheets were placed in an oven set at a temperature of 40 °C for 30 min to move moisture before fabricating the composites. Hand layup technique was implemented to manufacture 4 mm-thick laminates, which were achieved by using 6 sheets of unidirectional flax fibers. Epoxy resin was mixed and poured onto the fiber sheet until saturated and the sheets were then placed on top of each other to make the composites where the fiber volume ratio (V_f_) was maintained at 25%. This is based on a previous study, which showed that the weight proportion of the fiber content is in the range of 23% to 34% influenced by several factors including the resin and fiber type and the manufacturing process (hand layup and vacuum bagging processes) [30]. Our study lies in between. Similar findings have been reported in other works [31,32]. A metal roller was used to distribute the resin evenly over the fibers, ensuring good wettability of the fibers and to free any trapped air, as shown in Figure 1b. For the flax-reinforced composites with nanoparticles, different percentages of graphene—i.e., 0.5%, 1.0%, and 1.5% by weight—were mixed into the epoxy resin before being applied to the fibers. An electric shear mixer was used to ensure a homogeneous mix and to prevent aggregation of the graphene particles, as suggested by the graphene supplier. High-shear mixer facilitates the dispersion of graphene particles within the epoxy matrix by creating the shear force through the high-speed rotary motion of the mixer. This allows less aggregation and good dispersion of these nanoparticles in the epoxy matrix. The flax fibers moistened with resin were placed in the vacuum bag and sealed to start the vacuuming process at a constant pressure of 92 bar (see Figure 1c). This manufacturing technique was also implemented by Muralidhara et al. [33], Geren et al. [34], and Huang et al. [35] to produce high-quality composites. The manufactured sheets were left for 24 h under vacuum to cure initially and then post-cured for 3 h at 120 °C to enhance the heat distortion temperature (HDT) of epoxy (65 °C), as recommended by the manufacturer. The sheets were then cut to the required specimen dimensions for interlaminar shear and flexural tests.

### 2.3. Mechanical Testing

**Figure 2:**
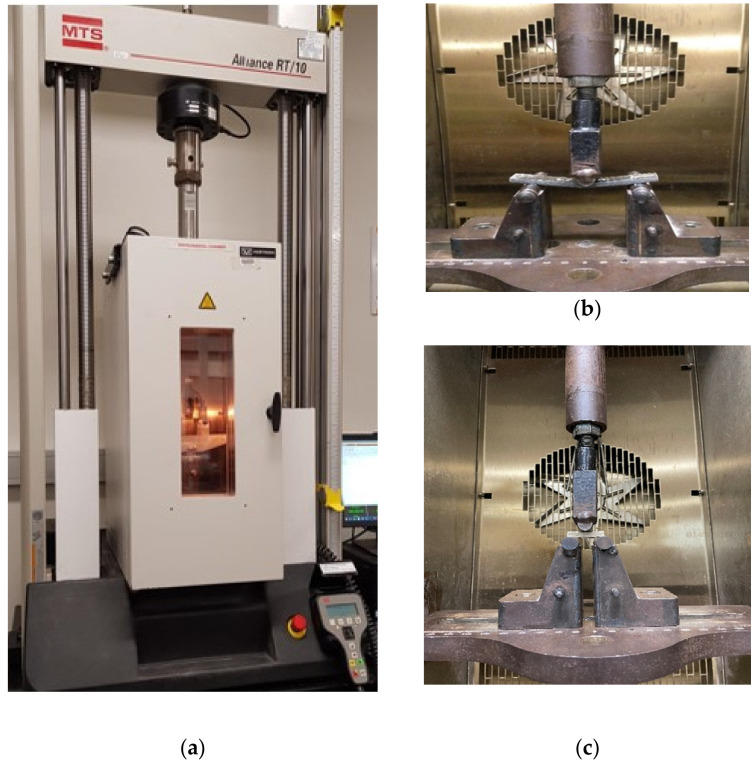
Mechanical tests for flax composites with various weights of graphene: (**a**) Instron Environmental Chamber; (**b**) Flexural Test; (**c**) Interlaminar Test.

The flexural and interlaminar shear properties of flax-fiber-reinforced epoxy composites with different graphene percentages under in-service elevated temperature were characterized following ASTM D790 [36] and ASTM D2344 [37], respectively. Table 2 shows the standard sample dimensions for different mechanical tests based on the ASTM standards and the required number of samples for each test. At elevated temperature, the specimens were tested using an Instron 3119 (Illinois Tool Works Inc., Norwood, MA, USA) environmental chamber (Figure 2a) at RT, 40, 60, 80, and 100 °C, where RT refers to the reference specimens tested at room temperature (20 °C). The specimens were preheated in the oven at the target exposure temperature for at least 45 min prior conducting the test to ensure consistent temperature throughout the thickness, as recommended by Alajarmeh et al. [38]. The test commenced once the chamber reached the target temperature and was maintained for 5 min. All test samples were performed in a steady state using the 100 kN MTS machine (see Figure 2b,c) at a loading rate of 1.3 mm/min.

### 2.4. Scanning Electron Microscope (SEM) Observation

The scanning electron microscope (SEM) JEOL JXA 840A (Tokyo, Japan) was used to examine the damage features, fracture surface, and fiber–matrix interface of the samples with and without graphene tested under different levels of temperature. SEM observations were also conducted to evaluate the distribution of the graphene nanoparticles within the composite matrix. The samples were carefully prepared by cutting around 10 mm by 10 mm from the tested specimens and then observed under the SEM.

## 3. Results and Observations

### 3.1. Flexural Behavior of Hybrid Flax Fiber-Reinforced Epoxy Composites

#### 3.1.1. Flexural Strength and Modulus under Elevated Temperature

**Figure 3:**
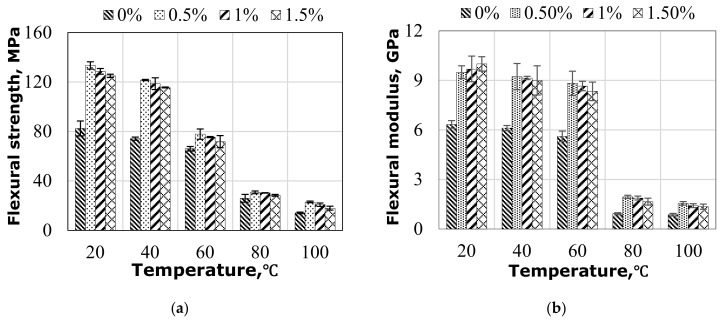
Temperature influence on the flexural properties of hybrid composites: (**a**) Flexural strength; (**b**) flexural modulus.

Figure 3 shows the flexural behavior of hybrid flax-fiber-reinforced epoxy composites with different graphene percentages at different levels of in-service elevated temperature. The values of 0%, 0.5%, 1.0%, and 1.5% in Figure 3 represent the amount of graphene nanoparticles by weight of the matrix. From Figure 3, significant improvements of flexural strength (FS) can be observed in composites with the addition of nanoparticles. It is worth mentioning that the highest average FS value at room temperature was obtained from the samples with 0.5% graphene (see Figure 3a). Adding more than 0.5% graphene, however, showed a consistently negative effect on the FS. The increase in temperature resulted in a significant reduction in the FS of the composites (Figure 3a), regardless of having graphene or not. This can be due to matrix softening. It was observed in Figure 3a that the composites without graphene showed a gradual decrease in the FS as the temperature increased to 60 °C. At a temperature above 60 °C, the FS of the composites dropped significantly. Beyond 8 °C, steady state in the FS was observed for all composite samples. This behavior can be explained by softening the matrix at higher temperatures. Since the test temperature is higher than the *T_g_* of the composite, indicating a change in the molecular chain movement of the epoxy matrix, the bonding strength of the fibers with synthetic resin deteriorates at elevated temperature. Thus, this bonding is unable to mediate load transfer from the matrix to the fibers and the role of graphene addition is insignificant, but some of the fibers that remain intact can bear some load at this temperature level. Figure 3b shows the effect of the amount of graphene on the flexural modulus (FM) of flax-fiber-based epoxy composites at elevated temperature. A significant improvement in FM can be observed when adding graphene nanoparticles. At RT, adding 0.5% of graphene increased the FM by 50.8% (see Figure 3b). However, increasing the graphene content by 1.0% and 1.5% briefly increased the FM by 2.1% and 5.3%. At elevated temperature, linear reductions of 11.4%, 7.1%, 10.7%, and 16.6% in the FM values until 60 °C can be observed in the tested samples with graphene contents of 0%, 0.5%, 1.0%, and 1.5%, respectively, compared with the samples tested at room temperature.

It is worth highlighting that the increase in graphene addition percentage indicates the high sensitivity of the composite samples with higher graphene content to elevated temperature due to other phenomena arising from the presence of graphene, which contributed to obtaining the trend of mechanical strength values for hybrid composites at elevated temperature. This trend of reduction in mechanical strength can be explained by the thermal effect from the vast differences in the thermal expansion coefficients of flax fiber, epoxy resin, and graphene particles in the hybrid composites. Flax fibers have a negative coefficient of thermal expansion in their longitudinal direction (−8 × 10^−6^/°C) [39]. As for graphene and its derivatives, thermal expansion coefficient ranges from −7 × 10^−6^ K^−1^ to −0.77 × 10^−6^ K^−1^ as reported by Gangineni et al. [40], while epoxy matrix has a thermal expansion coefficient of 64–68 × 10^−6^ K^−1^ [40]. This means that there is a difference between the thermal stress values developed at the interface layers reinforced with various materials. As such, the deterioration at the interface increases with increasing temperature at the presence of graphene and thus results in lower mechanical performance. At 80 °C and 100 °C, in contrast, samples with 0%, 0.5%, 1.0%, and 1.5% graphene content show almost a retention of the FM in a range of 14.6% to 16.3%. This is because composite samples with graphene recorded a *T_g_* temperature of 78 °C (for all samples with various graphene percentages), where the influence of the graphene addition becomes insignificant.

#### 3.1.2. Failure Behavior in Flexure under Elevated Temperature

**Figure 4:**
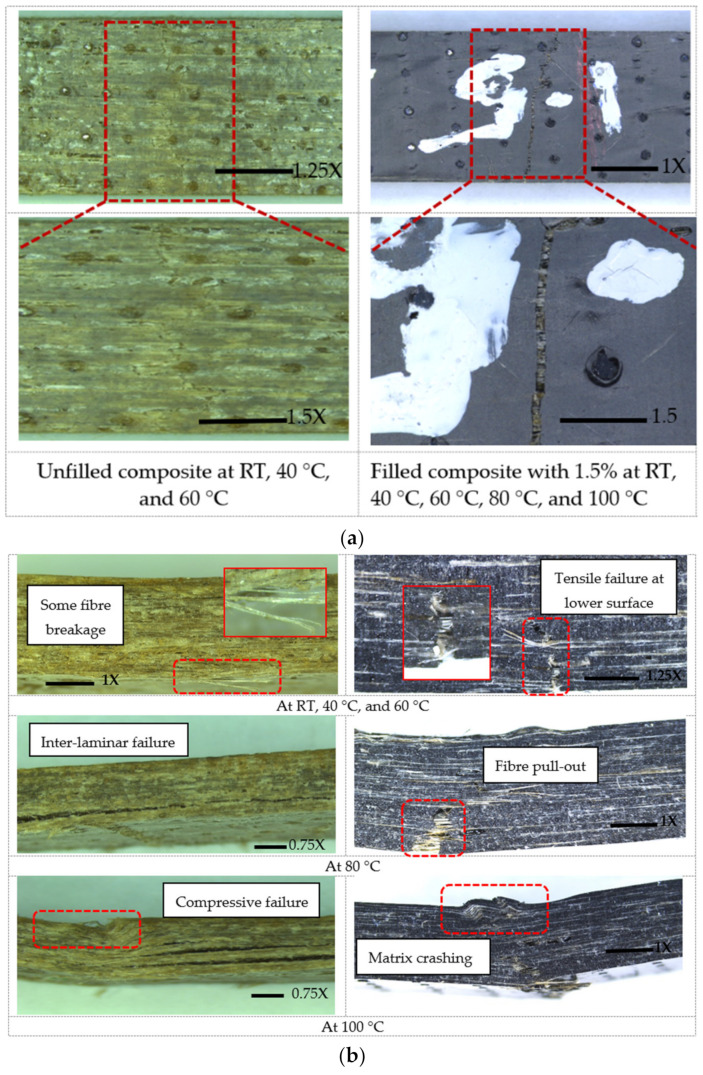
Flexural failure mechanisms for filled and unfilled composites at elevated temperature: (**a**) Tensile failures of hybrid composites with 0% and 1.5% at elevated temperature; (**b**) description of failure from the side view of the sample with 0% and 1.5%.

The failure modes in flexure of hybrid flax-fiber-reinforced epoxy composites under elevated temperatures are shown in Figure 4, which can be classified into four modes: (1) Tensile Failure (TF) at lower surface, (2) Fiber Breakage (FB), (3) Fiber Pull-out (FP), and (4) Interlaminar Failure (IF). All these modes of failure were accompanied by cracking in the matrix. The mechanisms of the different failure modes are described below.

TF: This failure occurs when the load stress applied on a sample exceeds the material’s strength [41]. This load stress causes individual fibers to break in the matrix. With an increase in the applied load, more fiber breakage occurs near the neighboring fractured fibers and then total tensile failure occurs. The tension region (lower surface) is the critical zone for the sample under these stresses. This failure was observed in specimens without graphene and tested at 20 °C, 40 °C, and 60 °C (see Figure 4a). This failure has also been observed for composites with graphene at different weight percentages and tested at levels of in-service elevated temperature. Due to this failure, a flexural crack intruded a few millimeters into the sample thickness (see Figure 4b) from the lower surface up towards the loading point.

FB: This failure occurs when local high stresses associated with the cracks of the cross-matrix and the intersection of the cracks of polymer matrix and ply interfaces occurred, as also observed by Jawaid et al. [42]. This means that when the fiber’s tensile strength is exceeded, the FB occurs. This was observed in specimens without graphene and tested at RT, 40 °C, and 60 °C (see Figure 4b). Hybrid nanocomposites tested at RT, 40 °C, 60 °C, 80 °C, and 100 °C also failed because of fiber breakages.

FP: This failure occurs when the interfacial bonding is weak, causing the fibers to slip out of the matrix. Exposure of the hybrid composites to the in-service elevated temperature caused softening of the epoxy matrix allowing the fibers to slide in the direction of flexural loading. This type of failure was observed in specimens with various graphene weight percentages and tested at 80 and 100 °C (see Figure 4b).

IF: Interlaminar failure is caused by the high interlaminar stresses developed between the layers of the fibers [43,44]. This failure is often described, as the separation of layers within composite laminates caused by matrix failure was observed in all specimens with graphene when tested at 100 °C and for composites without graphene when tested at 80 °C and 100 °C. The difference, however, is that the IF failure occurs parallel to the direction of reinforcement for composites without graphene (see Figure 4b), but this failure was propagated in an inclined plane through the sample thickness for composites with graphene (see Figure 4b).

### 3.2. Interlaminar Shear Behavior of Hybrid Flax-Fiber-Reinforced Epoxy Composites

#### 3.2.1. Interlaminar Shear Strength under Elevated Temperature

**Figure 5:**
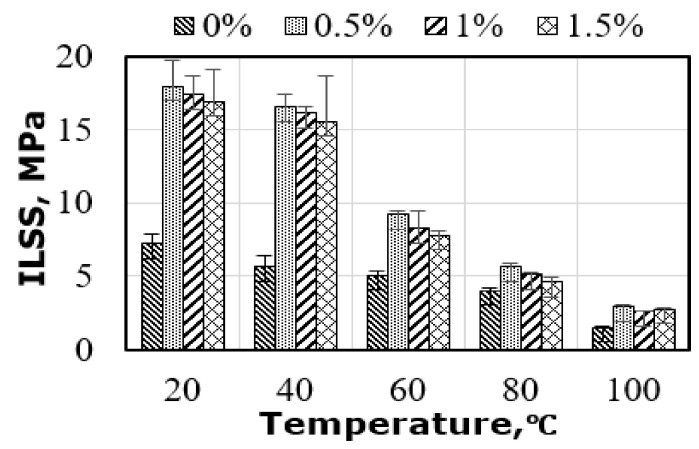
Relationship between temperature and ILSS results.

Figure 5 displays the effect of the amount of graphene along with temperature on interlaminar shear strength (ILSS) of flax-fiber-based epoxy composites. The addition of the nanoparticles revealed a significant increase in the ILSS of hybrid composites. The ILSS of the tested composites with 0.5%, 1.0%, and 1.5% graphene at room temperature increased by 148.6%, 138.9%, and 134.7%, respectively, when compared with the specimens without graphene. On the other hand, the composites without graphene showed a progressive and uniform reduction in the ILSS of around 15% for every level of temperature considered in this study. On the contrary, the composites with graphene showed only a 10% reduction in ILSS when the test temperature increased from RT to 40 °C. A 45% reduction in ILSS was then observed once the temperature was increased from 40 °C to 60 °C. A further reduction of 68% in ILSS was noticed when tested at 100 °C.

#### 3.2.2. Failure Modes in ILSS under Elevated Temperature

**Figure 6:**
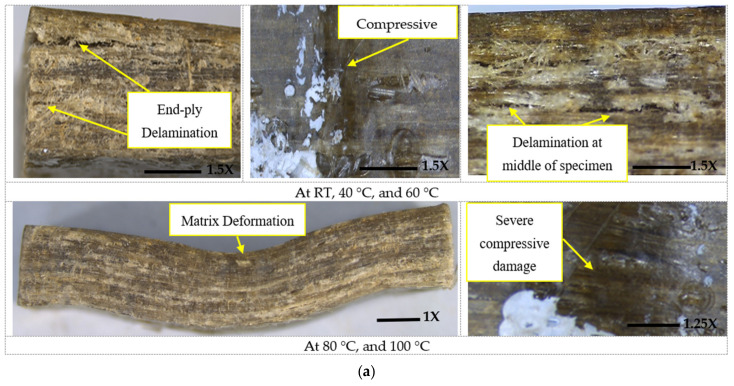

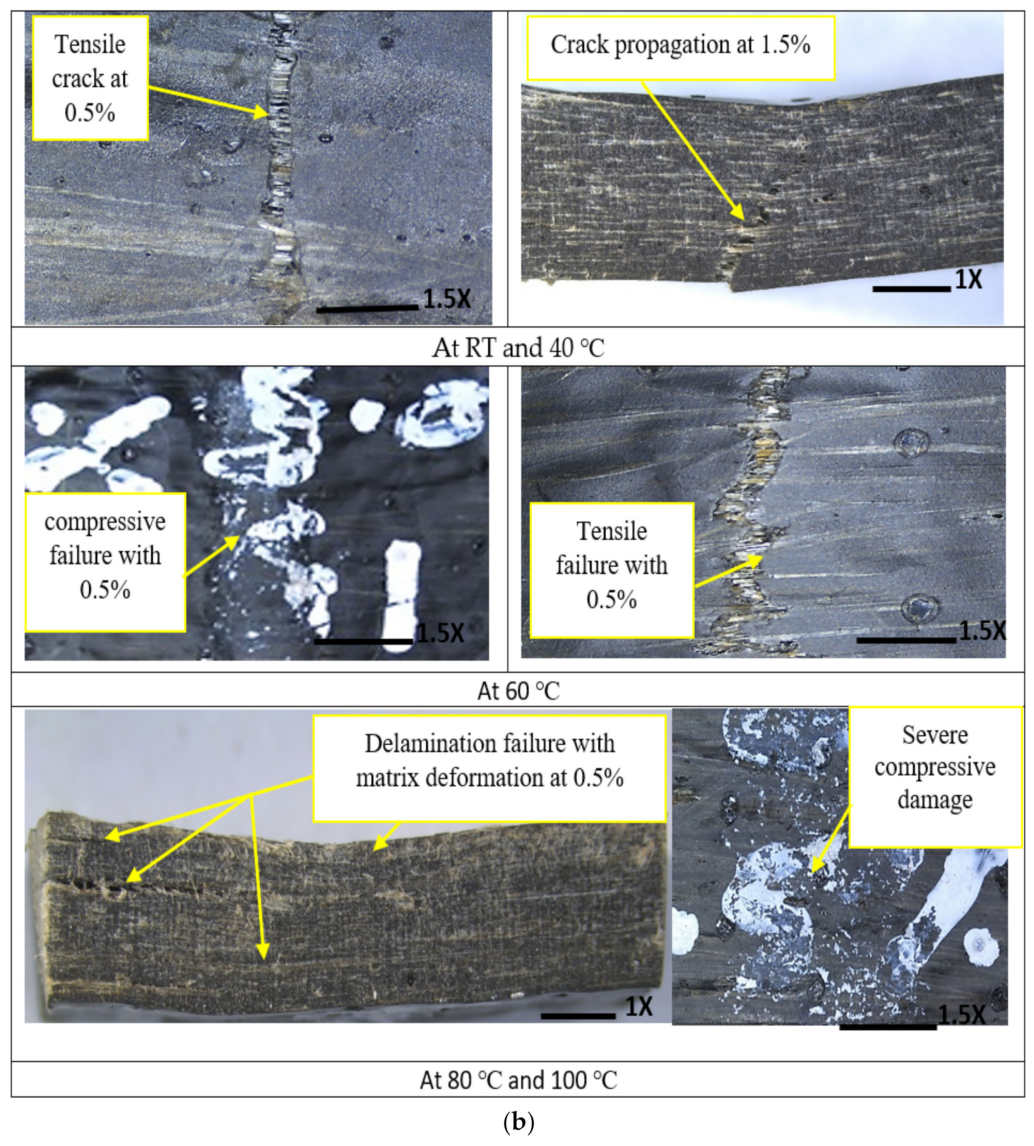
Short beam failure features under elevated temperature: (**a**) Failure mode of flax fiber composites; (**b**) failure mode of hybrid nanocomposites with 0.5% and 1.5%.

The failure modes of composites with and without graphene in ILSS under elevated temperature can be categorized as (1) End-ply Delamination (ED), (2) Compressive Failure (CF), (3) Matrix Deformation (MD), and (4) Mid-span Delamination (SD). A description of these failure modes is provided below:

- ED: This failure can be identified as a horizontal interlaminar crack initiated at the end of the specimens and propagated to the mid-span of the sample (Figure 6a). This failure occurs due to the composites exceeding their interlaminar shear strength at the ends due to the applied stress. ED was observed in specimens without graphene at RT, 40 °C, and 60 °C. This failure in samples with graphene changed to tensile failure at lower surface and distributed through the thickness in an inclined plane due to the increase in matrix stiffness caused by graphene addition at RT and 40 °C (see Figure 6b).
- CF: This type of failure occurs by compressive stresses on the upper surface and was observed in specimens without graphene when tested at different levels of temperature, as can be seen in Figure 6a. As for specimens with different levels of graphene by weight, compressive damage was observed in their top surface when tested at 60 °C, 80 °C, and 100 °C (see Figure 6b).
- MD: This mode of failure appeared as permanent deformation in the specimens without graphene nanoparticles and tested at 80 °C and 100 °C (see Figure 6a). This permanent deformation is accompanied with compression at the top surface for all the specimens with graphene and tested at 80 °C and 100 °C, as shown in Figure 6b. The tested composites failed by MD were able to retain a part of their original straightness after the removal of the applied load.
- SD: Increasing the temperature up to 80 °C and 100 °C for composites without graphene and with graphene resulted in failure by severe horizontal interlaminar cracks. SD failure initiated at the mid-span and propagated towards the supports because of the high mid-span deflection under loading, as shown in Figure 6a,b. This failure was also accompanied with matrix deformation under the loading point as it became soft at elevated temperatures.

### 3.3. SEM Image Analysis

**Figure 7:**
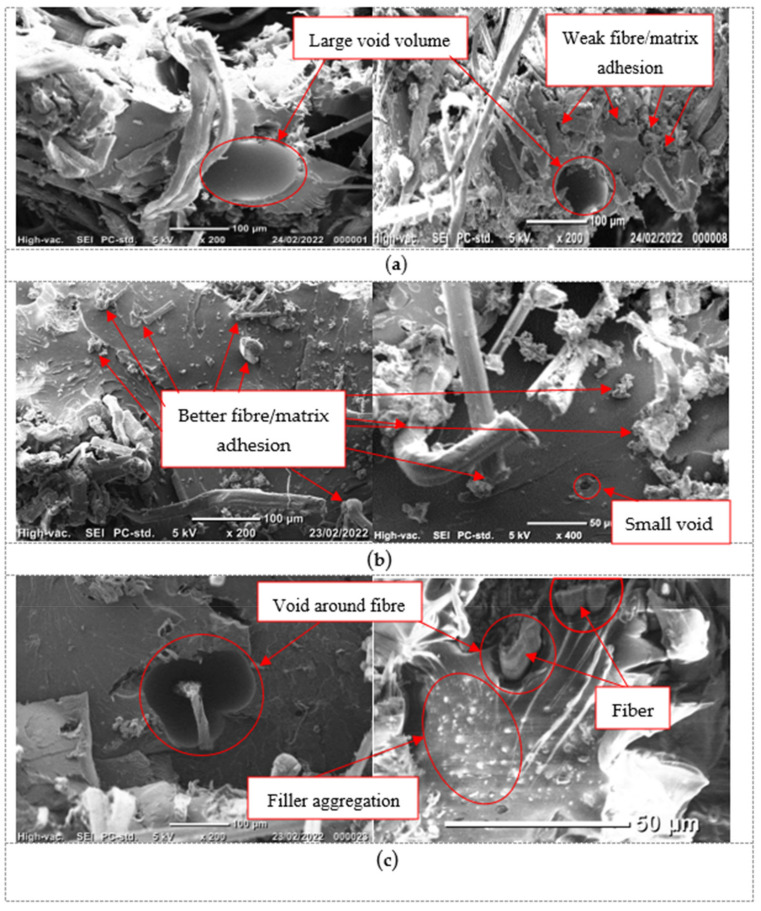

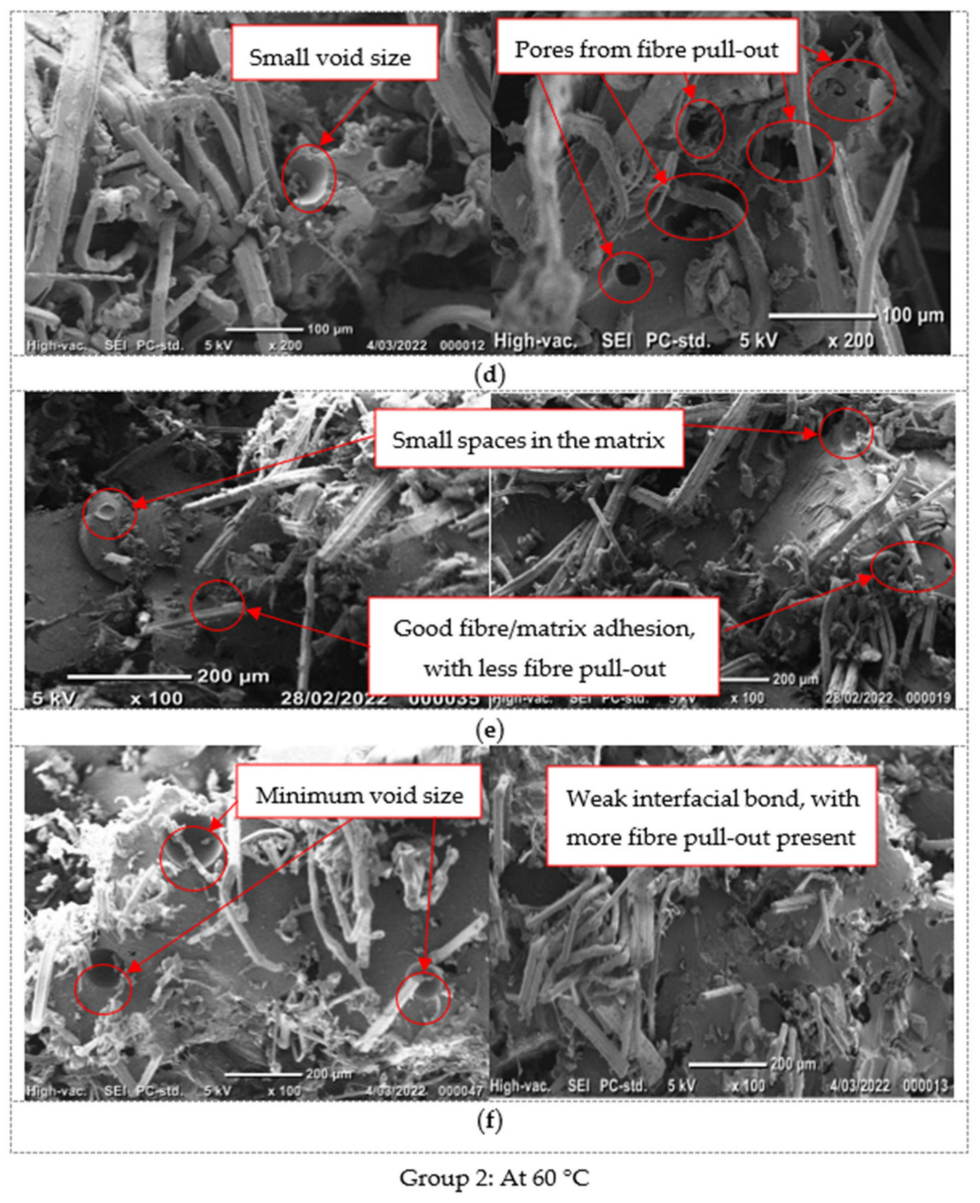
SEM images for specimens with various percentages of graphene: (**a**) Composite without graphene; (**b**) composite with 0.5%; (**c**) composites with 1% and 1.5% tested at RT; composites (**d**–**f**) with 0%, 0.5%, and (1%, 1.5%), respectively, tested at 60 °C.

The microstructure of flax fiber composites with various amounts of graphene at room and at elevated temperature was analyzed by Scanning Electron Microscope (SEM), as shown in Figure 7. The SEM image (up to magnification of ×500 times) also provided information of the internal structure showing the distribution of the fibers and the dispersion of the graphene to the epoxy matrix. It is worth mentioning that the analysis was conducted on the fracture surfaces of the tested specimens to evaluate the effect of the graphene and temperature on the observed failure modes.

Figure 7a shows large voids in the resin matrix of the control specimen, with weak fiber–matrix adhesion at room temperature. For specimens with 0.5% graphene, Figure 7b exhibits well-distributed and properly bonded graphene particles to the epoxy resin with a lower content of voids in the matrix where these voids were not around the fibers. Better fiber–matrix adhesion was also clear on these specimens, which was evident from the absence of fiber pull-out.

In contrast, the specimens with higher graphene percentages (1.0% and 1.5%) showed a greater number of void formations caused by filler agglomeration induced by the increase in the viscosity of the epoxy resin (see Figure 7c). These voids are clearly observed around the flax fibers, indicating that the fibers are not bonded properly to the epoxy resin at these locations. This also led to the formation of more weak points where failure can be initiated by fiber breakage. The formation of voids due to filler agglomeration in the filled composites contributes to poor interfacial bonding. This weakness in interfacial bonding can also explain the observed interlaminar failure of all composites. As such, increasing the viscosity by adding nanoparticles has been affirmed in other investigations [45].

At a higher temperature, the SEM images were chosen at 60 °C because this temperature is the HDT of the epoxy at which the resin begins to soften [26]. Further, according to the supplier instructions, the HDT of epoxy is 65 °C after 3 h curing time at 120 °C, as mentioned in the previous specimen preparation section. The *T_g_* for epoxy was also measured by DMA test, which was 64.6 °C. In the case of softening, although reducing the size of voids in all composites, regardless of filled or not, softening of the resin resulted in a continuous decrease in mechanical properties. For the control specimen tested at 60 °C, more pores were observed due to fiber pull-out, as shown in Figure 7d. The specimens with 0.5% graphene had good fiber–matrix interface adhesion, with less fiber pull-out present (see Figure 7e). However, specimens with 1% and 1.5% showed more fiber pull-out, indicating evidence of poor fiber–matrix interface adhesion (see Figure 7f).

## 4. Discussion

### 4.1. Influence of Amount of Graphene on Flexural Strength and Stiffness

Adding graphene nanoparticles to the resin matrix changed the flexural behavior of the flax–epoxy composites. For instance, adding 0.5% of graphene to the neat resin increased the flexural strength and modulus by 62% and 47%, respectively, at room temperature (Figure 3a,b). This increase in the flexural strength and modulus can be explained by the inclusion of graphene that filled the available space within the epoxy resin and improved the wettability of natural fibers and polymer matrix by building a bridge between them. This bridge facilitated the uniform distribution of individual fibers and reduced fiber–fiber interactions and fiber entanglement. The better adhesion between the fibers and resin led to a higher efficiency for transferring internal stresses through the interphase. This finding agrees with the results of the study as suggested by Kamaraj et al. [45], Zhou et al. [46], and Aswathnarayan et al. [47] wherein they observed an enhancement of flexural and tensile strength of plant fiber composites with the inclusion of graphene. They explained that the improvement in the mechanical properties was because of the uniform distribution of filler and improvement of interfacial interaction between the filler and the fibers. It is worth mentioning that graphene particles are stiffer materials than epoxy resin, which increases the stiffness of the matrix but makes it more brittle [48,49,50,51]. Increasing the percentage of the graphene to 1.0% and 1.5% slightly decreased the flexural strength by 3% and 4%, respectively, compared with the 0.5% addition. The mode of failure was similar, wherein the composites failed by a compressive crack at the top and a tensile crack at the bottom and with fiber breakage at the cracked zone due to the agglomeration and voids’ formation upon increasing the graphene due to the epoxy becoming more viscous, as observed in Figure 7. These defects caused localization and premature failure, as shown by the observed failure where fiber breakage occurred mostly for those fibers passing through the voids (see Figure 7c). As expected, adding more graphene nanoparticles increased the stiffness of the matrix resulting in composites with higher flexural strength and modulus than the specimens without graphene.

### 4.2. Influence of Amount of Graphene on ILSS

The addition of graphene significantly enhanced the interlaminar properties of the flax fiber–epoxy composites. Adding 0.5% of graphene increased the ILSS by 250% compared with the composites without graphene because of the stronger fiber–matrix interface developed by adding nanoparticles, which allowed stress to transfer easily across the fibers. This was supported by the observed failure behavior, wherein the composites with 0.5% graphene failed by flexural failure (Figure 6b) and not by interlaminar shear. Increasing the graphene to 1.0% and 1.5% slightly reduced the failure stress by 3% and 5%, respectively, compared with the 0.5% addition because of filler agglomeration. These phenomena can be explained by the increased viscosity of epoxy resin because of the increase in the percentage of graphene, which leads to more agglomeration of the filler. This small reduction in ILSS at room temperature is similar to the results reported by Koirala et al. [52], in which carbon nanotube (CNT) sheets reinforced polymer composites showed a gradual reduction in ILSS at room temperature. They explain that this behavior is a result of CNT agglomeration. It should also be mentioned that all the specimens with graphene addition showed flexural failure at room temperature (Figure 6b).

### 4.3. Influence of Elevated Temperature on Flexural Strength and Stiffness

**Figure 8:**
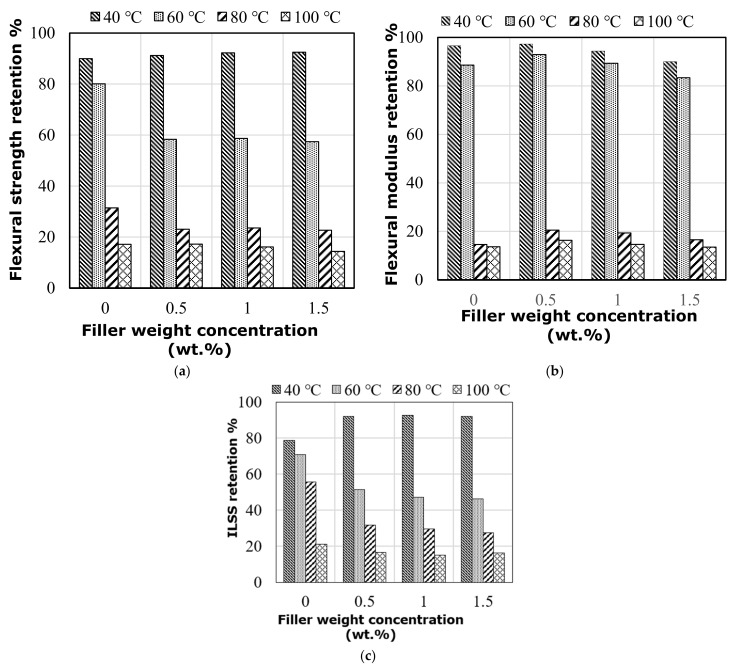
Property retention of the tested specimens at elevated temperature: (**a**) flexural strength retention; (**b**) flexural modulus retention; (**c**) interlaminar shear strength retention.

The flexural strength and stiffness of flax-fiber–epoxy composites with graphene are affected differently at in-service elevated temperatures. At 40 °C, the flexural strength and modulus decrease by around 7–10% and 3–5%, respectively, for all samples, regardless of whether they are filled or not, as shown in Figure 8a,b. There is also no effect of the mode of failure (see Figure 4b). At 40 °C, nevertheless, the increase in the graphene percentage (0.5%, 1.0%, and 1.5%) resulted in higher flexural strength and modulus retention. This means that resin at this temperature started to soften but is still able to hold the fiber and graphene together and transfer stress between them. However, increasing the service temperature to 60 °C reduced the flexural strength and modulus by (20%, 42%, 42%, and 43%) and (15%, 19%, 18%, and 17%) for the specimens with (0%, 0.5%, 1.0%, and 1.5%), respectively. Flexural strength started to be affected by this level of test temperature whereas the flexural modulus remained stable, as the former property was mostly controlled by the interface between the fiber and matrix and the latter property was governed by the properties of the fiber. It can be clearly observed that at this temperature, the resin came close to the *T_g_*, indicating the start of its softening. Moreover, the specimens with graphene showed higher degradation loss in strength under elevated temperature than the specimens with no graphene, which was attributed to the extreme variation in the thermal expansion coefficients of the three phases that developed different internal thermal stresses at the interface of the samples with graphene. This resulted in a lower efficiency of transferring internal stresses through the interface of specimens with graphene than specimens without graphene particles. Unlike the findings observed at 40 °C, increasing the percentage of graphene particles at 60 °C briefly reduced the retention in flexural strength of the tested specimens, confirming the effect of graphene anisotropy on the thermal stresses developed at the interface when increasing the amount of graphene. In other words, the extent of thermal expansion was higher in the hybrid composites with more graphene, leading to further deterioration of the interface. A significant reduction in the flexural properties has been witnessed at 80 °C and 100 °C (see Figure 8a,b). At 80 °C, specimens with no graphene showed 31% and 14% retention in flexural strength and modulus, respectively, whereas specimens with graphene showed almost 22% and 20% retention in flexural strength and modulus, respectively. Furthermore, at 100 °C, specimens with no graphene showed 17% and 11% retention in flexural strength and modulus, respectively. Similarly, specimens with graphene showed only 15% and 14% retention in flexural strength and modulus, respectively, indicating the insignificant effect of graphene on the mechanical performance at high service temperature.

### 4.4. Influence of Elevated Temperature on ILSS

The composites with and without graphene particles behaved differently when tested in ILSS at elevated temperature. The ILSS reduction in the specimens with no graphene particles was 22% compared with the 8% reduction in those with graphene particles at 40 °C (see Figure 8c). Due to the graphene inclusion, the latter specimens showed flexural failure instead of the interlaminar failure observed by the former specimens. In contrast, even when showing the same mode of failure at 60 °C, specimens with graphene percentages of 0.5%, 1.0%, and 1.5% revealed 52%, 47%, and 44% retention in the failure load compared with the specimens tested at room temperature. On the other hand, the specimens with no graphene showed 70% retention in the ILSS value. As mentioned earlier, this finding refers to different thermal expansion coefficients between three phases in the hybrid composite, which resulted in various thermal stresses at the interface when tested at elevated temperature leading to a higher reduction in mechanical properties. Moreover, it was obvious that specimens without graphene retained higher ILSS at 80 °C and 100 °C (57% and 20%, respectively) compared with the ones with graphene at the same temperatures (30% and 16%, respectively) because of the absence of graphene at the interface of flax fiber composite, and the extent of thermal expansion was less at the interface. Moreover, with the higher thermal stresses in the latter specimens resulting from the development of different thermal expansion between the layers reinforced with different types of reinforcements, their mode of failure changed from flexural to severe interlaminar shear (Figure 6b), which revealed a significant reduction in ILSS retention compared with the unfilled specimens.

## 5. Conclusions

This study investigated the effect of graphene nanoparticles on the flexural and interlaminar shear strength (ILSS) properties of flax fiber epoxy composites at in-service elevated temperature. Hybrid composites with graphene ranging from 0, 0.5, 1.0, and 1.5% by weight of epoxy resin were prepared and tested at a temperature of 20 °C to 100 °C with increments of 20 °C. From the test results and observations, the following points are concluded:

An increase in the flexural and ILSS strength of flax fiber–epoxy composites at room temperature was achieved with the addition of graphene nanoparticles. The addition of 0.5% graphene increased the flexural and ILSS strength of tested composites by 62% and 149%, respectively, due to the increase in bond strength at interface between the epoxy resin and flax fibers caused by the contribution of graphene nanoparticles. Increasing the amount of graphene from 0.5% to 1.0% and 1.5%, however, reduced the flexural strength and ILSS strength by 57% and 52%, and 142% and 135%, respectively, attributed to the filler agglomeration as witnessed from the SEM observations.

- The addition of graphene nanoparticles improved the flexural modulus of flax fiber composites by 50%, 53%, and 58%, respectively, for 0.5%, 1.0%, and 1.5% at room temperature. This improvement in the flexural stiffness resulted in the failure mode changing from ductile to a brittle manner due to the greater stiffness of the graphene.
- The addition of graphene nanoparticles has an insignificant influence on the flexural and ILSS strength of flax fiber composites at in-service elevated temperature. The flexural and ILSS strength retention of the composites with 0.5% graphene nanoparticles were 60% and 52%, respectively, at 60 °C, and only 18% at 100 °C. This is a result of the high difference in the thermal expansion of the hybrid composites with graphene, which contributes to the increase in thermal stresses and their effect on the bonding strength of the interface at in-service elevated temperature. In contrast, the composites without graphene retained their flexural strength and ILSS up to 80% and 60%, respectively, at 60 °C, with similar strength retention to the composites with graphene at 100 °C.
- The mode of failure in flexural samples with graphene changed from the fiber breakage at room temperature to fiber pull-out at elevated temperature showing that the matrix properties governed the failure at in-service elevated temperature. Similar failure behavior can be observed for flexural test specimens without graphene at room temperature. Under ILSS, the stronger fiber–matrix interface by adding graphene nanoparticles changed the failure behavior from interlaminar shear to tensile failure and fiber pull-out at low and moderate temperature, and from fiber pull-out and tensile failure to delamination failure at elevated temperature.

It can be recommended that further studies are required to investigate the inclusion of graphene into natural fiber composites considering the durability and environmental behavior of this type of composite. Various types of filler can be also investigated to study the bonding mechanism between different fillers and fibers.

## Figures and Tables

**Table 1:** Properties of flax fibers and neat epoxy resin.

Material	Density (g/cm^3^)	Elastic Modulus (GPa)	Tensile Strength (MPa)	Reference
Flax fibers	1.40	70	1400	[28]
Epoxy resin	1.12–1.17	3.4	130	Technical Data Sheet [29]

**Table 2:** Test specimen geometry and a number of coupons based on ASTM standard for evaluating mechanical properties.

Type of Test	Standard	No. of Coupons	Dimensions (mm)		
			Length	Width	Thickness
Flexural test	ASTM D790:2007	5	80	16	4
Interlaminar test	ASTM D2344:2016	5	24	16	4

## Data Availability

All the data required are reported in this manuscript.
